# Segmental motion at the peak of the ossification foci is independent risk factor except for mal-alignment and thick ossification foci for poor outcome after laminoplasty for cervical ossification of the posterior longitudinal ligament: analyses in patients with positive K-line, lordotic alignment, and lower canal occupying ratio

**DOI:** 10.1186/s13018-020-01903-3

**Published:** 2020-09-14

**Authors:** Junya Saito, Masao Koda, Takeo Furuya, Satoshi Maki, Yasushi Ijima, Mitsuhiro Kitamura, Takuya Miyamoto, Sumihisa Orita, Kazuhide Inage, Fumio Hasue, Takayuki Fujiyoshi, Koshiro Kamiya, Yoshikazu Ikeda, Fumitake Nakajima, Mitsuhiro Hashimoto, Hiroshi Noguchi, Hiroshi Takahashi, Masashi Yamazaki, Seiji Ohtori

**Affiliations:** 1 Department of Orthopedic Surgery, Chiba University School of Medicine, 1-8-1 Inohana Chuou-ku Chiba City, Chiba, 260-8670 Japan; 2 Department of Orthopedic Surgery, University of Tsukuba, Tennodai, Tsukuba, Ibaraki 305-8575 Japan; 3 Department of Orthopedic Surgery, Kimitsu Chuo Hospital, 1010, Sakurai, Kisarazu, Chiba, 292-8535 Japan; 4 Department of Orthopedic Surgery, Chiba Rosai Hospital, 2-16, Tatsumidaihigashi, Ichihara, Chiba, 290-0003 Japan; 5 Department of Orthopedic Surgery, Toho University Chiba Medical Center, 564-1, Shimoshizu, Sakura, Chiba, 285-8741 Japan

**Keywords:** Ossification of the posterior longitudinal ligament, K-line, Laminoplasty, Risk factor, Segmental range of motion

## Abstract

**Purpose:**

To elucidate the independent preoperative factors that have a significant impact on poor surgical outcome after laminoplasty for K-line (+) ossification of the posterior longitudinal ligament (OPLL). Analyses in K-line (+) patient population can exclude the influence by mal-alignment and thick OPLL, both of which are well known two major factors that have significant impact on clinical outcome.

**Methods:**

The present study included 72 patients (50 male and 22 female) who underwent laminoplasty for K-line (+) cervical OPLL and were followed-up for at least 1 year. Recovery of Japanese Orthopedic Association score (JOA score) for cervical myelopathy was used as the measure of clinical outcome. For radiographic assessment, the type of OPLL, the maximum OPLL occupation ratio, the C2-C7 angle, and the segmental range of motion at the peak of OPLL (segmental ROM) were assessed. To elucidate the factors that are significantly associated with a poor clinical outcome after laminoplasty for K-line (+) OPLL, statistical analyses were conducted.

**Results:**

The mean preoperative JOA score was 8.9 points and improved to 12.8 points after surgery. The recovery of JOA score was 47 ± 35%. Stepwise logistic regression following univariate analyses revealed that preoperative segmental ROM at the peak of OPLL is an independent factor associated with a poor outcome (*p* = 0.04, odds ratio = 1.15).

**Conclusions:**

Large preoperative segmental ROM at the peak of the OPLL is an independent factor that has significant impact on poor surgical outcome after laminoplasty for K-line (+) OPLL.

## Introduction

In Japan, expansive laminoplasty is widely applied to ossification of the posterior longitudinal ligament (OPLL) of the cervical spine, because it can achieve simultaneous multilevel spinal cord decompression, and does not need any special procedures or techniques. The rational for spinal cord decompression by laminoplasty for anteriorly located OPLL is a bow-stringing effect, which achieves posterior shift of the spinal cord by removal of the posterior element of the spinal canal based on lordosis of the cervical spine [1]. Therefore, OPLL patients with kyphosis and/or thick ossification foci may have an unfavorable outcome because the bow-stringing effect cannot work sufficiently in such patients [2, 3]. To predict sufficient decompression by laminoplasty preoperatively, the K-line was conceived [4]. The K-line is defined as an imaginary line between the midpoints of the spinal canal at C2 and C7. If the peak of the OPLL does not exceed the K-line, which means lordosis and/or ossification foci are not so extensive, the patient will be categorized as K-line (+), and sufficient spinal cord decompression can be obtained by laminoplasty alone. By contrast, in patients with the peak of OPLL exceeding the K-line, which means kyphosis and/or ossification foci are extensive, then the patient will be categorized as K-line (–), and insufficient decompression by laminoplasty alone is predicted. K-line (–) OPLL patients show poor surgical outcome after laminoplasty alone [5]. Therefore, anterior decompression and fusion surgery or posterior decompression with instrumented fusion surgery should be applied for K-line (–) OPLL patients.

Analyses in K-line (+) patient population can exclude the influence by mal-alignment and thick OPLL, both of which are two major factors that have significant impact on clinical outcome. The primary aim of the present study was to elucidate the independent preoperative factors that have a significant impact on poor surgical outcome after laminoplasty for K-line (+) OPLL, omitting the possible influence by mal-alignment and thick OPLL.

## Methods

The present study included 72 patients (50 male and 22 female) who underwent laminoplasty alone for K-line (+) cervical OPLL and were followed-up for at least 1 year. Therefore, the present study excluded K-line (–) patients with kyphosis and/or thick ossification foci, both of which are the major known risk factors for insufficient decompression by laminoplasty alone. The average age at surgery was 66.5 years (range 31–86 years) and the mean follow-up period was 52.6 months (range 12–194 months). The demographic data of the patients is shown in Table 1.

**Table 1:** Patient demographics

Patient demographics	(*n* = 72)
Male: Female	50: 22
Age at surgery (years old)	66.5 (31-86)
Follow-up period (month)	52.6 (12-194)
Japanese Orthopedic Association (JOA) score (points)	
Pre-op.	8.9 (2-15)
Post-op.	12.8 (5.5-17)
Recovery rate	46.6 ± 35.0%

Recovery of Japanese Orthopedic Association score (JOA score) for cervical myelopathy [6] was used as the measure of clinical outcome. The recovery of JOA score was calculated using the following formula: percentage recovery = ((postoperative JOA score−preoperative JOA score)/(17−preoperative JOA score)) × 100 [7].

**Fig. 1:**
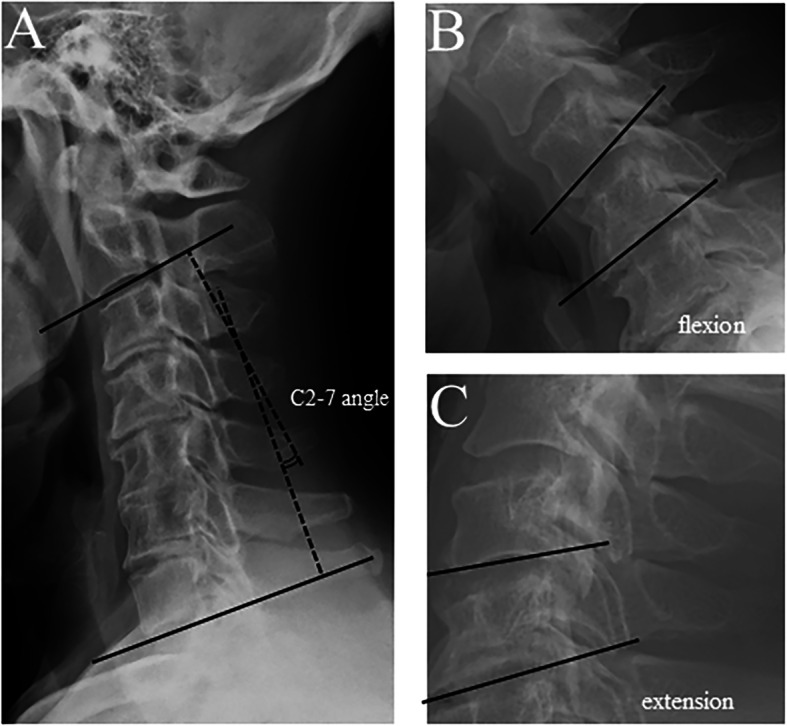
Measurement of C2-7 angle and range of motion. C2-7 angle was measured as angle between C2 and C7 lower endplates in lateral cervical spine X-ray in neutral position (**a**). Range of motion was measured as chage in angle between corresponding spinal segment in cervical spine X-ray in flexion and extension position (**b**)(**c**)

Radiographic assessments were performed as follows: the type of OPLL was classified into continuous, segmental, and mixed type according to the classification described by Tsuyama et al. [8]. The maximum OPLL occupation ratio was defined as the thickness of the peak of OPLL divided by the anteroposterior diameter of the bony spinal canal on lateral plain X-ray images of the cervical spine (Fig. 1). The C2-C7 angle was measured by the angle between the C2 and C7 inferior endplates in pre- and postoperative lateral radiographs of the cervical spine, and was expressed as positive values for lordosis and negative values for kyphosis. Preoperative segmental range of motion (segmental ROM) was expressed as an increase in the angle between the inferior endplates of the vertebrae at the peak of OPLL in the flexion–extension lateral radiograph.

To elucidate the factors that are significantly associated with a poor clinical outcome after laminoplasty for K-line (+) OPLL, statistical analyses were conducted as follows. Patients who showed JOA score recovery less than the average of all the patients were considered to have poor surgical outcome. Correlation between a poor surgical outcome and all patient factors (including age, sex, and preoperative JOA score) and imaging assessments described above (including type of OPLL, maximum OPLL occupation ratio, preoperative C2–C7 angle, preoperative segmental ROM) were analyzed using univariate analysis. Factors that showed a *p* < 0.1 were selected for further analyses. Next, the selected factors were subjected to logistic regression analyses using a stepwise method to determine which independent factors were significantly associated with a poor surgical outcome after laminoplasty for K-line (+) OPLL. Receiver-operator characteristic (ROC) curve analyses were performed to determine the cutoff value predicting a poor outcome. Values are expressed as mean ± SD. All the analyses were conducted using JMP (version 10.0.2; SAS Institute, Cary, NC, USA).

## Results

The mean preoperative JOA score was 8.9 points (range 2 to 15) and improved to 12.8 points (range 5.5 to 17) after surgery. The recovery of the JOA score was 47 ± 35% (Table 1).

The types of OPLL were classified into continuous (1 case), mixed (39 cases), segmental (27 cases), and local (5 cases) types. The average maximum occupation ratio of the OPLL was 44.7 ± 11.8%. The average preoperative segmental ROM at the peak of the OPLL was 5.2 ± 3.9°. The average preoperative C2–C7 angle was 11.5 ± 8.8° (Table 2).

**Table 2:** Imaging analyses

Imaging analyses	
Type of ossification of the posterior longitudinal ligament (OPLL)	
Continuous	1 case
Mixed	39 cases
Segmental	27 cases
Local	5 cases
Maximum occupation ratio (%)	44.7 ± 11.8
Pre-op. C2-7 angle (degree)	11.5 ± 8.8
Pre-op. segmental range of motion (ROM) at peak of OPLL (degree)	5.2 ± 3.9

By univariate analyses, the maximum occupation ratio of the OPLL and preoperative segmental ROM at the peak of OPLL showed a significant correlation with poor surgical outcome after laminoplasty alone for K-line (+) OPLL. Stepwise logistic regression revealed that preoperative segmental ROM at the peak of OPLL is an independent factor associated with a poor outcome (*p* = 0.04, odds ratio = 1.15), whereas the other factor we examined, maximum occupation ratio of the OPLL (*p* = 0.07), was not significant. ROC analyses showed that the cut-off value of preoperative segmental ROM at the peak of the OPLL to predict poor surgical outcome was 4.2°, and the area under the curve value was 0.67, showing a moderate predictability (Table 3). Thus, if the preoperative segmental ROM is greater than 4.2°, poor surgical outcome after laminoplasty is expected.

**Table 3:** Univariate analysis and stepwise logistic regression

	*p* value
Univariate analysis	
Age	0.24
Sex	0.21
Follow-up period	0.36
Pre-op. JOA score	0.24
Type of OPLL	0.68
Maximum occupation ratio	0.09^#^
Pre-op. C2-7 angle	0.29
Pre-op. segmental ROM at peak of OPLL	0.03^#^
Stepwise logistic regression	
Pre-op. segmental ROM at peak of OPLL	0.04*
Maximum occupation ratio	0.07

*JOA* Japanese Orthopedic Association, *OPLL* ossification of the posterior longitudinal ligament, *ROM* range of motion

^#^*p* < 0.1

*p < 0.05

## Discussion

We found that a large preoperative segmental ROM at the peak of the OPLL is an independent factor having a significant impact on poor surgical outcome after laminoplasty for K-line (+) OPLL.

In the present study, we only included patients with K-line (+), excluding K-line (–) patients who have kyphosis and/or thick ossification foci, both of which are well known major deteriorative factors after laminoplasty. Kyphosis and thick ossification foci inhibit sufficient posterior spinal cord shift caused by bow-stringing effect, resulting in insufficient decompression after laminoplasty. This suggested that the present series of patients would obtain sufficient spinal cord decompression by laminoplasty alone. A large preoperative segmental ROM at the peak of OPLL can impede neurological recovery even though sufficient decompression is obtained by laminoplasty.

There are several previous reports describing this impediment. Masaki et al. reported that patients showing a poor surgical outcome had a significantly greater preoperative segmental ROM at the peak of OPLL. They concluded that anterior decompression and fusion surgery is suitable for such patients [9]. Maruo et al. reported that the C2-C7 ROM of patients with a poor surgical outcome after laminoplasty for OPLL was significantly greater than that in patients with a better outcome [10, 11]. These lines of evidence suggest that a large ROM at the peak of the OPLL can impede neurological recovery after laminoplasty. However, definitive conclusion cannot be drawn from those previous reports because those reports include heterogenic patient population, mal-alignment, and thick OPLL, both of which are the major confounding factors on clinical outcome after decompression surgery for OPLL. Possible advantages of the present study is the homogeneity of the patients population by enrolling only K-line (+) patients, which means good lordotic alignment and relatively lower canal occupying ratio, contributing to exclude the confounding factors.

Possible mechanisms underlying the impact of a large segmental ROM on poor neurological recovery are as follows. First, the spinal cord can be impinged at the peak of OPLL, because the peak of OPLL could be an apex during cervical spine flexion, even after sufficient posterior decompression achieved by laminoplasty. Next, there is a possibility that preoperative spinal cord impingement because of large preoperative segmental ROM could induce repetitive minor trauma to the spinal cord, which might result in irreversible spinal cord tissue damage. We cannot distinguish between these two mechanisms. Further investigation is needed to elucidate this issue. We are now applying short segmental fusion to laminoplasty if the preoperative segmental ROM is relatively large. Future comparison between laminoplasty alone and laminoplasty with short segmental posterior fusion for K-line (+) OPLL with large segmental ROM at the peak of the OPLL will elucidate the mechanisms underlying the impact of large segmental ROM on neurological recovery after laminoplasty.

## Conclusions

Large preoperative segmental ROM at the peak of the OPLL is an independent factor that has a significant impact on poor surgical outcome after laminoplasty alone for K-line (+) OPLL. Therefore, conversion to anterior surgery or addition of instrumented fusion to laminoplasty should be considered for such patients.

## Data Availability

The datasets generated during and analyzed during the current study are available from the corresponding author on reasonable request.

## Abbreviations

OPLL: Ossification of the posterior longitudinal ligament
JOA: Japanese Orthopedic Association
ROM: Range of motion
ROC: Receiver-operator characteristic
SD: Standard deviation
